# Respiratory Muscle Strength Training in Parkinson’s Disease—A Systematic Review and Meta-Analysis

**DOI:** 10.3390/healthcare13101214

**Published:** 2025-05-21

**Authors:** Irene Navas-Garrido, Javier Martín-Núñez, Julia Raya-Benítez, María Granados-Santiago, Alba Navas-Otero, Laura López-López, Marie Carmen Valenza

**Affiliations:** 1 Department of Physiotherapy, Faculty of Health Sciences, University of Granada, Av. De la Ilustración, 60, 18071 Granada, Spain; ing97@correo.ugr.es (I.N.-G.); javimn@ugr.es (J.M.-N.); cvalenza@ugr.es (M.C.V.); 2 Nursing Department, Faculty of Health Sciences, University of Granada, Av. De la Ilustración, 60, 18071 Granada, Spain; juliarb@ugr.es (J.R.-B.); mariagranados@ugr.es (M.G.-S.); 3 Department of Physiotherapy, Faculty of Health Sciences, University of Málaga, Arquitecto Francisco Peñalosa, 3, Campanillas, 29071 Málaga, Spain; albanavas@ugr.es

**Keywords:** Parkinson’s disease, respiratory muscle training, peak expiratory flow rate, maximal respiratory pressures

## Abstract

**Background/Objectives:** The aim of this review was to evaluate the effectiveness of respiratory muscle strength training in patients with Parkinson’s disease (PD). **Methods**: A systematic review and meta-analysis of randomised controlled trials (RCTs) was performed on PubMed, Web of Science, and Scopus databases. We included RCTs that evaluated the effectiveness of respiratory muscle training in patients with PD versus no intervention, sham treatment, or a different type of intervention. Quality assessment and risk of bias were assessed using the Downs and Black scale and the ROB2 tool. **Results**: Finally, 10 studies were included. The methodological quality of the studies was “good” in most of the studies, with results ranging from 21 to 25. In terms of risk of bias, six of them indicated low risk and four of them showed unclear risk of bias. Data were pooled and a meta-analysis of maximum expiratory pressure (MEP), maximum inspiratory pressure (MIP), and voluntary peak expiratory flow rate (PEFR) was performed. Meta-analysis indicated a significant overall effect of respiratory muscle strength training on MEP (MD = 17.08; 95% CI = 2.32, 31.84; *p* = 0.02) and on voluntary PEFR (MD = 1.50; 95% CI = 0.51, 2.48; *p* = 0.003). However, results in the meta-analysis showed a non-significant overall effect of respiratory muscle strength training on MIP (MD = 1.69; 95% CI = −11.91, 16.29; *p* = 0.82). **Conclusions**: The synthesis of evidence presented in this systematic review and meta-analysis underscores the potential of respiratory muscle strength training as an effective means of increasing MEP and PEFR in patients with PD.

## 1. Introduction

Parkinson’s disease (PD) is the most common neurodegenerative movement disorder [1]. The prevalence of this pathology has doubled in the last 25 years [2]. In 2019, over 8.5 million people suffered from PD, but it is estimated that this number will increase to 9 million people globally by 2030 [3].

The disease generally occurs in advanced age individuals, but younger persons can also be affected. The male gender is affected more frequently than females [2] and it is characterised by an extensive and selective loss of nigrostriatal dopaminergic neurons.

The main PD-associated manifestations are tremor, rigidity, bradykinesia/akinesia, postural instability, and gait difficulty [4]. However, it includes numerous non-motor symptoms [5] such as cognitive decline, pain, behaviour changes, sleep disorders, and autonomic dysfunction.

The related central and peripheral effects of PD influence the neuromuscular system, showing muscle weakness and loss of movement control that can have a negative impact on the respiratory system [6].

Abnormal functioning of the respiratory system is found in Parkinson’s disease, primarily stemming from respiratory muscle rigidity, weakness, and a lack of coordinated contraction for maximal efforts [7,8], alongside chest wall stiffness and limited cough airflow [9,10]. All of this significantly contributes to the swallowing and phonation issues experienced by over 89% of individuals with PD throughout the course of their ailment [11,12]. These difficulties are further linked to peripheral muscle disability and overall respiratory impairment, highlighting the interconnected nature of motor and respiratory dysfunction in the progression of Parkinson’s.

These symptoms are not particular to subjects in advanced stages. Research has shown that mildly affected individuals also have important decreases in respiratory muscle strength and this is a significant cause of morbidity and disability [13,14]. Over time, these symptoms get worse and result in elevated rates of mortality [15].

Accordingly, respiratory assessment has been underscored as a factor to consider in the comprehensive care of PD.

The medical handling of PD is widely studied. It involves levodopa that improves the motor and non-motor symptoms of this disease, although the observed enhancements normally vary based on dopaminergic administration. It depends on whether patients are in the ‘on’ or ‘off’ state, with poorer results during the ‘off’ state.

However, medical treatment alone is not sufficient to improve the respiratory condition of these patients. Thus, respiratory muscle training (RMT) has emerged as an option in rehabilitation plans with the purpose of meliorating the breathing function and quality of life of the PD population. It is based on the fundament that respiratory muscles have the ability to react to workout stimuli via modifications in their structure comparable to those happening in the rest of the skeletal muscles [16].

RMT is a technique that aims to improve the strength, endurance, and overall function of the muscles responsible for breathing (inspiratory and expiratory muscles) through specific and repeated exercises. It involves targeted exercises designed to overload these muscles, similar to how you would train skeletal muscles, to make them stronger and more efficient [17].

Previous studies have evidenced benefits on respiratory function after RMT (including inspiratory and expiratory muscle training) in neuromuscular diseases like multiple sclerosis [18] and after stroke [19].

Systematic reviews have previously investigated the effects of respiratory muscle training (RMT), including inspiratory and expiratory muscle training, in PD [20,21]. These reviews suggest potential benefits for respiratory muscle strength, swallowing function, and phonatory aspects. They also highlight the need for further research to standardise training protocols. Our study addresses a critical gap in the existing literature by conducting the first meta-analysis on this topic [20,21].

This quantitative synthesis of available data offers a novel and statistically robust evaluation of the overall impact of RMT on respiratory function in PD. By pooling the results of individual studies, our meta-analysis aims to provide a more precise and reliable estimate of the treatment effect than can be gleaned from individual trials or qualitative reviews alone. This is particularly important for informing clinical practice and future research directions in the management of respiratory complications in PD.

For this reason, the aim of this review was to evaluate the effectiveness of inspiratory and expiratory muscle strength training in patients with Parkinson’s disease (PD).

## 2. Methods

### 2.1. Protocol and Registration

This systematic review and meta-analysis were performed following the Preferred Reporting Items for Systematic Reviews and Meta-Analyses (PRISMA) [22] statement guidelines (Supplementary File S1: PRISMA checklist). The protocol for this systematic review was registered with PROSPERO (International Prospective Register of Systematic Reviews) under the registration number CRD42018108358.

### 2.2. Search Strategy

A systematic search of articles was conducted on PubMed, Web of Science, and Scopus databases from inception to April 2025. The search strategy was carried out based on the investigation of keywords used in existing systematic reviews, such as the terms “respiratory training”, “inspiratory muscle strength training”, “expiratory muscle strength training”, “respiratory muscle rehabilitation”, “parkinson’s disease”, and “parkinsonism”. In addition, we screened the references of relevant reviews for further articles that can be potentially included. Table 1 describes the search strategy used in each database.

Criteria for considering studies for this review were based on the PICOS [23] model (participants, interventions, comparisons, outcome, and study design):

P (Participants): adults with Parkinson’s disease.

I (Intervention): respiratory muscle strength training that requires the use of a specific device.

C (Comparison): any other rehabilitation therapy, placebo, sham, or no treatment group.

O (Outcomes): respiratory muscle strength, quality of life, phonatory measures, pulmonary function, swallowing function, cough, and peak flow parameters.

S (Study Design): randomised clinical trials (RCT).

Only full-text, RCT written in English and Spanish were included. Grey literature, clinical practice guidelines, systematic reviews and meta-analyses, abstracts, letters, editorials, theses, dissertations, observational studies, clinical practice guidelines, and conference papers were excluded.

The initial search was carried out by two independent reviewers (I.N.G and L.L.L), who were responsible for identifying and eliminating duplicate records, as well as screening titles, abstracts, and full texts deemed potentially relevant. To minimise the risk of selection bias, any disagreements were discussed and resolved in consultation with a third reviewer (J.M.N). Only after the final selection of studies was completed did the team proceed with data extraction and the assessment of methodological quality. Data were extracted using structured templates containing predefined fields. These fields included citation details (author and publication year), participant demographics (such as age, sex, and disease severity), intervention types (e.g., inspiratory or expiratory muscle strength training, sham interventions), dopaminergic medication status (on/off), training parameters (including frequency, intensity, and duration), follow-up periods, measured outcome variables, and the corresponding results.

The methodological quality of the included studies was assessed using the Downs and Black checklist [24], which comprises 27 items distributed across five domains: reporting quality, external validity, internal bias, confounding (selection bias), and statistical power. Based on the total score, studies were categorised as follows: scores ≥26 indicated “excellent” quality, 20–25 as “good,” 15–19 as “fair,” and ≤14 as “poor.” Additionally, the risk of bias was evaluated using the Cochrane Risk-of-Bias Tool for Randomized Controlled Trials (ROB2) [25], which examines five key areas: randomisation procedures, adherence to intended interventions, completeness of outcome data, outcome measurement methods, and selective reporting. Each study was then rated as having a low, high, or unclear risk of bias.

### 2.3. Meta-Analysis

Quantitative analyses were performed using Review Manager 5 (Rev-Man version 5.1, updated March 2011) for all studies that reported post-intervention means and standard deviations for maximum inspiratory pressure (MIP), maximum expiratory pressure (MEP), and peak expiratory flow rate (PEFR). Relevant data, such as final mean values, standard deviations, and the number of patients assessed at different time points in each treatment group, were extracted to compute overall mean differences between groups. Continuous outcomes were analysed using weighted mean differences when studies used the same measurement scale. If different scales were employed to assess the same underlying condition or symptom, standardised mean differences were calculated. For each outcome, 95% confidence intervals were calculated. To estimate the overall effect size, random-effects or fixed-effects models were used, depending on the results of the I^2^ test for heterogeneity. An I^2^ value < 50% was considered indicative of low heterogeneity, in which case a fixed-effects model was applied [26]. A visual inspection of forest plots was also conducted to identify any outlier studies.

## 3. Results

### 3.1. Study Selection

**Figure 1:**
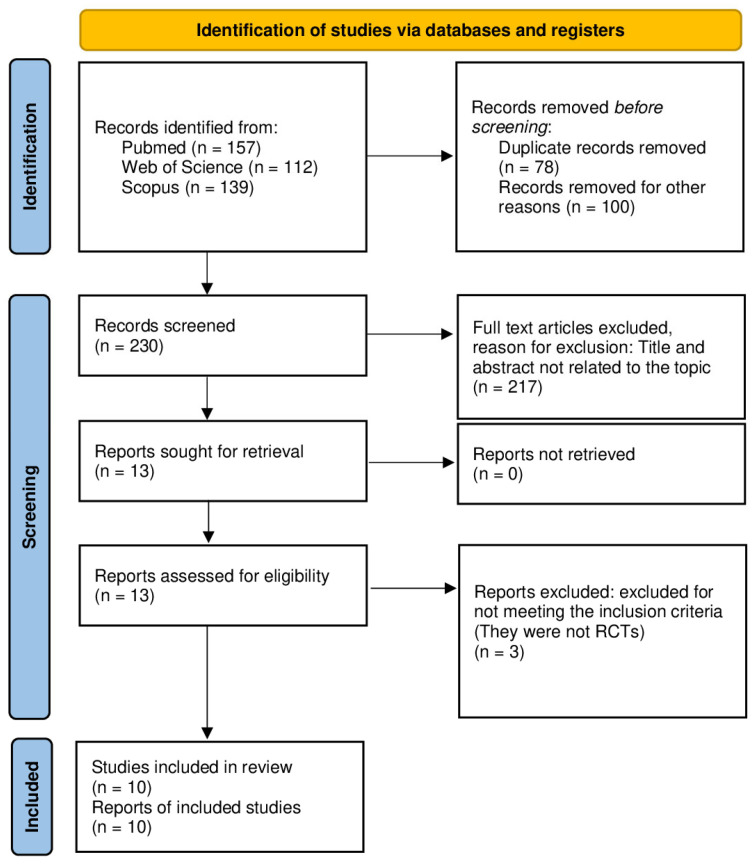
PRISMA flow chart.

A flow diagram of the search, screening, and selection process is shown in Figure 1. The initial search identified 408 studies. After removing duplicates, 330 articles were obtained. Screening based on the title and abstract resulted in the selection of 34 records. From these 34 studies, 24 articles were excluded following the evaluation of the full text; no potentially eligible studies were excluded on the basis of language during the selection process. At last, a total of ten studies [27,28,29,30,31,32,33,34,35,36] were included in the qualitative syntheses, and four [29,30,32,34] were incorporated into the quantitative synthesis.

### 3.2. Study Characteristics

Table 2 presents a summary of the characteristics of the included studies and their main findings.

As seen in Table 2, the number of participants ranges from nine to sixty. Most of them were advanced-aged men, between 57.3 and 70.45 years old. Parkinson’s disease severity was between I and IV according to the Hoehn and Yahr scale, and the range of time since diagnosis was from 5 to 8.58 years, although most of the studies did not report it. Studies investigated the effects of EMST, IMST, or a comparison between both of them. Interventions were homebased and their duration ranged from 4 to 12 weeks, with five to six sessions per week. Three different devices were handled for EMST and IMST: Threshold^®^ (Philips Respironics, USA), POWERbreathe^®^ (Southam, Warwickshire, UK), and EMST150 (Aspire products LLC., USA). Intensity was set from 50% to 75% of MEP and from 5% to 75% of MIP. Participants’ adherence was monitored through telephone follow-ups, once-weekly visits, or self-reported diaries. In half of the studies, participants were in an “on” state of dopaminergic medication at the time of assessments.

The most frequent outcomes were expiratory muscle strength measured by MEP; inspiratory muscle strength measured by MIP; peak expiratory flow rate (PEFR) and peak cough flow (PCF) measured in voluntary and reflex ways; and pulmonary function measured by forced vital capacity (FVC), forced expiratory volume in 1 s (FEV1), and slow vital capacity (SVC). To a lesser extent, some variables related to swallowing and phonation were studied.

Among the included articles, four studies compared EMST to sham treatment [28,31,34,35] and one compared EMST to sensorimotor training for airway protection (smTAP) [29]. One article compared IMST to sham treatment [36], another one contrast IMST to incentive spirometer therapy [30], and finally, one compared EMST to therapeutic singing [27]. In addition, two studies included three groups, EMST, IMST, and sham treatment group [32,33].

### 3.3. Main Findings

Of the studies that compared an EMST programme with sham treatment [28,31,34,35], all analysed expiratory muscle strength measured by MEP. In all of them, significant differences were found between the two groups in favour to the intervention group.

In addition, three of them [27,31,35] analysed quality of life and found no significant differences between the two groups.

In the study by Troche et al. conducted in 2023 [29], which compared a group that followed an EMST programme with another group that carried out smTAP, significant differences were observed in expiratory muscle strength, in favour of the EMST group. However, the smTAP group achieved higher PEFR and cough volumes than the EMST group.

In contrast, in the studies by Reyes et al. conducted in 2018 and 2020 [32,33], patients following an EMST programme obtained better PEFR values than the IMST group and the control group.

On the other hand, in the study by Mohammed et al. [30], a group following an IMST programme was compared with another group following a volume incentive inspirometer (VII) treatment plan. The results showed significant differences between the two groups in favour of the IMST group in inspiratory muscle strength measured by MIP. Inzelberg et al. [36] compared an IMST programme with a control group, where the results were along the same lines as the previous study, finding significant differences between the two groups, in favour of the IMST group.

In both studies, there were no statistically significant differences between the IMST group and the control group in terms of lung function. Along the same lines is the study by Reyes et al. in 2018 [33], where the results showed that the effect size was small for both the EMST and IMST groups, as well as for the control group in lung function variables (FVC and FEV1). However, in the study by Inzerlberg et al. [36], the group that undertook an IMST programme improved their perception of dyspnoea compared to the control group; and in the study by Mohammed et al. [30], the IMST group improved their exercise capacity compared to the control group.

In the articles that compared a group that followed an EMST programme with a group that followed an IMST programme and with a control group [32,33], variables such as expiratory muscle strength were studied, where the effect size was larger in the EMST group compared with the control group. In inspiratory muscle strength, when comparing the IMST group with the control, the effect size for this variable was larger in the IMST group.

The studies included in this review also incorporated other outcomes, such as phonatory and swallowing-related outcomes.

Specifically, the study by Reyes et al. in 2020 [32] analysed maximum subglottic pressure (SGP) and maximum phonation time (MPT) and found that the effect size was moderate between the IMST group and the control group; in contrast, if we compare the IMST group with the control group, the effect size was small. However, the study by Antonsson et al. [28] showed no significant differences in MPT between baseline and post-intervention measurements in the group of patients who underwent an EMST programme.

### 3.4. Methodological Quality and Risk of Bias Assessment

Table 3 shows the methodological quality scores.

The methodological quality of the studies was “good” for most of them with results ranging from 21 to 25 when the Downs and Black quality tool was used. Only three of them obtained 18 points and had “fair” quality.

**Figure 2:**
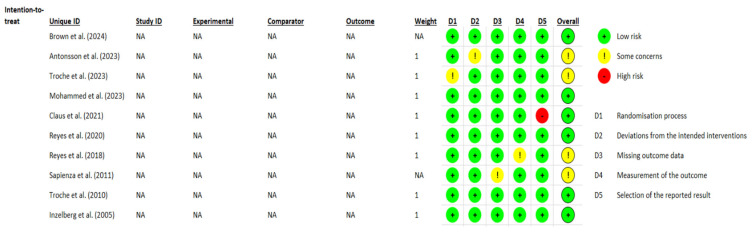
Cochrane Risk-of-Bias Tool version 2.0 scores. Brown et al. (2024) [27], Antonsson et al. (2023) [28], Troche et al. (2023) [29], Mohammed et al. (2023) [30], Claus et al. (2021) [31], Reyes et al. (2020) [32], Reyes et al. (2018) [33], Sapienze et al. (2011) [34], Troche et al. (2010) [35], Inzelberg et al. (2005) [36].

When the ROB2 was applied, six of them indicated low risk and four of them showed unclear risk of bias. ROB2 results are shown in Figure 2.

### 3.5. Results Obtained in Meta-Analysis

**Figure 3:**
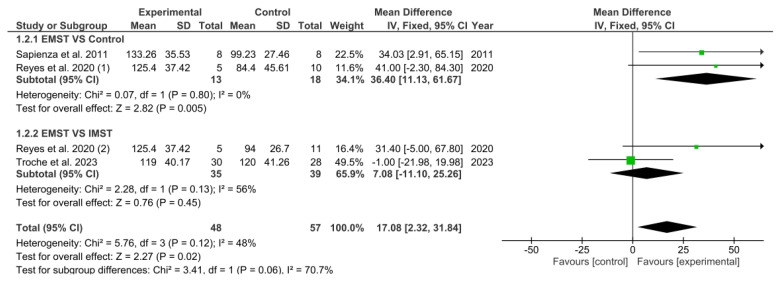
Results of MEP. Sapienza et al. 2011 [34], Reyes et al. 2020 (1) [32], Reyes et al. 2020 (2) [32], Troche et al. 2023 [35]. Green squares indicate the mean differences for each study; black diamonds represent the pooled mean difference with 95% confidence intervals.

As illustrated in Figure 3, the meta-analysis showed a significant overall effect of EMST on MEP, as evidenced by the pooled mean difference (MD). This difference was significant when compared to the comparator groups (MD = 17.08; 95% CI = 2.32, 31.84; *p* = 0.02).

A subgroup analysis was performed. The primary objective of the first subgroup was to ascertain whether EMST produced better results than the control group. The meta-analytic pooled MD indicated a significant overall positive effect of EMST when contrasted with the control group (MD = 36.40; 95% CI = 11.13, 61.67; *p* = 0.005). The second subgroup aimed to determine whether performing EMST was better than performing IMST to improve MEP. The pooled MD showed a non-significant overall effect of EMST compared with IMST (MD = 7.08; 95% CI = −11.10; 25.26; *p* = 0.45).

**Figure 4:**
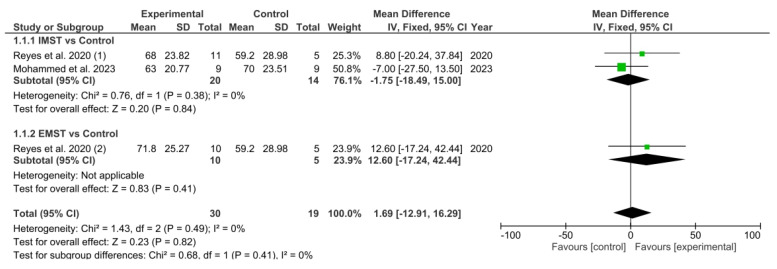
Results of MIP. Reyes et al. 2020 (1) [32], Mohammed et al. 2023 [30], Reyes et al. 2020 (2) [32]. Green squares indicate the mean differences for each study; black diamonds represent the pooled mean difference with 95% confidence intervals.

Figure 4 shows the results obtained in the meta-analysis concerning MIP. The pooled MD showed a non-significant overall effect of respiratory muscle strength training compared with the control groups (MD = 1.69; 95% CI = −11.91, 16.29; *p* = 0.82).

A subgroup analysis was performed. The initial subgroup sought to ascertain whether IMST resulted in better outcomes than the control group. However, the meta-analytic pooled MD indicated no significant overall effect of IMST (MD = −1.75; 95% CI = −18.49, 15.00; *p* = 0.84). Similarly, the second subgroup aimed to determine if EMST led to improved results compared to the control group, but the pooled MD also showed no significant overall effect (MD = 12.60; 95% CI = −17.24, 42.44; *p* = 0.41).

**Figure 5:**
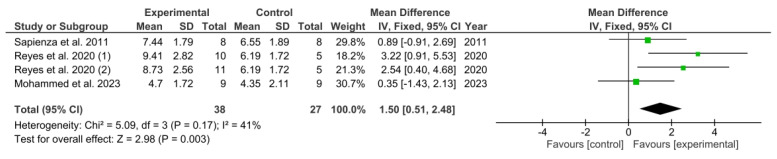
Results of voluntary PEFR. Sapienza et al. 2011 [34], Reyes et al. 2020 (1) [32], Reyes et al. 2020 (2) [32], Mohammed et al. 2023 [30]. Green squares indicate the mean differences for each study; black diamonds represent the pooled mean difference with 95% confidence intervals.

The findings from the meta-analysis regarding voluntary PEFR were examined, as illustrated in Figure 5. The calculated pooled MD indicated a significant overall impact of respiratory muscle strength training in comparison to the control group (MD = 1.50; 95% CI = 0.51, 2.48; *p* = 0.003).

## 4. Discussion

This systematic review and meta-analysis aimed to evaluate the effectiveness of respiratory muscle training in patients with PD.

The sample of this systematic review reflects the characteristics of the PD population. The reviewed studies only included patients with PD, except for Antonsson et al. [28], who also analysed another group of patients with multiple sclerosis.

Of the total patients, 65.7% were men, and the age ranged from 57.3 to 70.45 years; the data are consistent with the characteristics of this population, as PD has been shown to be more prevalent in men and older people [37].

The respiratory muscle training programmes were heterogeneous in terms of duration of intervention, but none lasted less than 4 weeks and all were carried out at home. The most repeated parameters were sets and repetitions performed per session, which in most studies were five by five, respectively.

The majority of the studies carried out EMST [27,28,29,31,34,35], two of them carried out IMST [30,36], and two studies carried out both of them [32,33].

The maximum intensity set in EMST was 75% of the MEP in all studies; however, for IMST it was variable depending on the study. These data are consistent with other training programmes conducted in PD patients in other reviews [21,38].

The results of this review indicate that respiratory muscle training may be an effective strategy for improving respiratory muscle strength in people with PD. Those who followed an EMST have obtained significant improvements in MEP, phonatory measures, dysphagia, and swallow safety. On the other hand, those who have carried out IMST have shown significant improvements in MIP and exercise capacity. Finally, articles that followed both of them (IMST and EMST) have shown significant improvement in MIP, MEP, and peak flow.

The meta-analysis showed a significant increase in voluntary PEFR and MEP compared to placebo or other interventions. These findings suggest that respiratory muscle training could be an intervention to consider in the management of upper airway protective mechanism disorders, typical of these patients.

Systematic reviews conducted to date that attempt to clarify whether respiratory muscle training is an effective tool for the management of patients with PD show similar results to those found in this systematic review [39]. In general, the results show that respiratory muscle strength training improves MEP, MIP, and PEFR in this type of patient.

However, the number of previously conducted reviews that exclusively included PD patients is very limited, most included other neuromuscular diseases, incorporated both randomised controlled studies and non-randomised controlled trials, and the intervention groups combined respiratory muscle training with other treatments.

Therefore, an updated review of all randomised controlled studies conducted to date that only included patients with PD and whose intervention was respiratory muscle training alone was needed. Furthermore, to our knowledge, this is the only review of respiratory muscle strength training and PD that has been able to include a meta-analysis.

The results obtained in our systematic review and meta-analysis are in line with previous systematic reviews, showing that respiratory muscle training had a positive effect on MEP and voluntary PEFR, which is considered an effective measure of airway clearance [40]. Silva et al. 2019 [18] carried out a Cochrane review with the aim of evaluating the effectiveness of RMT (including inspiratory and expiratory muscle training) in patients with neuromuscular disease. Although the authors advise caution when considering the conclusions, they concluded that maximum inspiratory and expiratory pressures improved respiratory muscle strength. In addition, Watson et al. 2022 [39] concluded that respiratory muscle training improves lung volumes and respiratory muscle strength in neuromuscular disease.

However, although our results shows that this type of training significantly improved MIP in PD patients, the meta-analysis showed a non-significant overall effect between the intervention group and the control group.

In the case of MIP, one of the possible explanations is that the control group performed a different type of intervention. In the study performed by Mohammed et al. [30], the control group used a volume incentive inspirometer (VII).

Previous studies have shown that inspiratory muscle strength increases after VII use due to increased recruitment of motor units [41,42]. It should be noted that when patients use VII and perform breathing exercises, they have to mobilise a considerable tidal volume together with a low respiratory rate, which presumably results in an increase in respiratory muscle strength due to the increased inhalation/exhalation ratio. This would probably explain why both groups improve their respiratory strength.

Furthermore, authors such as Reyes et al. [32,33] have pointed out that the baseline values of maximum respiratory pressures in this type of patient were lower in MEP than in MIP, which could suggest that in patients with PD, the inspiratory musculature would be less affected than the expiratory musculature. Therefore, the margin for improvement in MIP would be smaller. This, together with the fact that the control group underwent another intervention, could explain why the training effect was not significant in the group that performed an IMST programme.

In addition to the reasons described above, we must take into account that patients with PD have a number of limitations just because they have the disease. Between 28 and 94% of individuals with PD have a restrictive breathing pattern [43]. Respiratory muscle training involves synchronised contraction of the diaphragm, abdominal, and intercostal muscles, which are essential for proper rib cage and abdominal expansion.

The restrictive pattern hinders the normal functioning of the respiratory system by causing stiffness of the rib cage, reduced lung volumes, and loss of coordination of the respiratory muscles to contract during maximal efforts. These characteristics can be an obstacle to significant improvements.

On the other hand, none of the studies included in this review have reported adverse effects when performing this type of intervention, so we can consider that respiratory muscle training is safe.

### Limitations

This study is not without limitations. The results cannot be generalised to patients with advanced PD since most of the participants who were included in the different articles analysed were between stages I and III according to the Hoehn and Yahr scale. Future studies should focus on later stages of the disease, where more impaired pulmonary functions are observed. Another limitation is the small sample size of the studies, although there are some that have found a significant effect of EMST in this type of patient despite this [32,33].

Moreover, in most of the studies there was no follow-up, so it is unknown if the benefits obtained after treatment continue in the medium and long term. The use of a maintenance programme has been suggested by previous studies, which reported a decrease in MEP after a period without training in people with PD [34].

It should also be noted that in half of the studies included in this review, a history of dopaminergic medication was not taken into consideration when recruiting participants. Previous studies have shown a significant improvement in lung volumes and flows, as well as in respiratory muscle strength in patients receiving levodopa [32]. The option of training in the “on” state should be considered, since it contributes to improving the patients’ symptoms.

The training programme was homebased in all studies and was measured by patient records. These consisted of self-reported diaries and telephone follow-ups, which for the most part may be unreliable. Future research should monitor training and measure adherence to treatment in a more accurate way.

Another limitation has been the limited information available on respiratory muscle training in patients with PD. Very few studies met the inclusion criteria of this review; most of them mixed training with other interventions and were not RCT.

It would have been interesting to meta-analyse other variables, for example, related to swallowing and phonation, since these are affected in 89% of people with PD [31]. In the end, this has not been possible due to the lack of sufficient data to carry it out. Studies such as that of Troche et al. have indicated that EMST increases swallowing safety in PD patients with dysphagia [29].

Despite its limitations, this review shows that respiratory muscle training is an effective tool for increasing respiratory muscle strength in patients with PD.

However, the results should be considered with caution. Further studies are needed to understand the long-term effects of respiratory muscle training in this type of population. Future research should include larger sample sizes and follow-up periods to determine the effects of detraining in order to design appropriate maintenance programmes.

## 5. Conclusions

This systematic review and meta-analysis provide data on the efficacy of IMT and EMT for the improvement of respiratory muscle strength in PD. The results showed that IMT and EMT increase respiratory muscle strength and peak expiratory flow rate, which are essential to ensure the proper functioning of the upper airway protective mechanism in these patients. However, caution is required when interpreting the results due to the observed heterogeneity and the limited number of included studies. Further studies are needed to identify which training protocols are suitable to improve respiratory strength in these individuals. Despite these limitations, IMT and EMT appears as a promising option to improve respiratory muscle strength in patients with PD.

## Figures and Tables

**Table 1:** Search strategy used according to the database.

Database	Search Equation	Results
PubMed	((“respiratory training” OR “respiratory training intervention” OR “respiratory muscle strength training” OR “respiratory muscle strength programme” OR “respiratory muscle training programme” OR “respiratory training protocol” OR “respiratory muscle strengthening” OR “training of the respiratory musculature” OR “expiratory muscle strengthening” OR “inspiratory muscle strengthening” OR “inspiratory muscle training” OR “expiratory muscle training” OR “expiratory muscle strength training” OR “inspiratory muscle strength training” OR “programmes of expiratory and inspiratory muscle training” OR “breathing exercises” OR “inspiratory muscle rehabilitation” OR “expiratory muscle rehabilitation” OR “respiratory muscle rehabilitation”) AND (“parkinson disease” OR “parkinsonism” OR “parkinson’s disease”))	157
Web of Science	TS = ((“respiratory training” OR “respiratory training intervention” OR “respiratory muscle strength training” OR “respiratory muscle strength programme” OR “respiratory muscle training programme” OR “respiratory training protocol” OR “respiratory muscle strengthening” OR “training of the respiratory musculature” OR “expiratory muscle strengthening” OR “inspiratory muscle strengthening” OR “inspiratory muscle training” OR “expiratory muscle training” OR “expiratory muscle strength training” OR “inspiratory muscle strength training” OR “programmes of expiratory and inspiratory muscle training” OR “breathing exercises” OR “inspiratory muscle rehabilitation” OR “expiratory muscle rehabilitation” OR “respiratory muscle rehabilitation”) AND (“parkinson disease” OR “parkinsonism” OR “parkinson’s disease”))	112
Scopus	TITLE-ABS-KEY ((“respiratory training” OR “respiratory training intervention” OR “respiratory muscle strength training” OR “respiratory muscle strength programme” OR “respiratory muscle training programme” OR “respiratory training protocol” OR “respiratory muscle strengthening” OR “training of the respiratory musculature” OR “expiratory muscle strengthening” OR “inspiratory muscle strengthening” OR “inspiratory muscle training” OR “expiratory muscle training” OR “expiratory muscle strength training” OR “inspiratory muscle strength training” OR “programmes of expiratory and inspiratory muscle training” OR “breathing exercises” OR “inspiratory muscle rehabilitation” OR “expiratory muscle rehabilitation” OR “respiratory muscle rehabilitation”) AND (“parkinson disease” OR “parkinsonism” OR “parkinson’s disease”))	139

**Table 2:** Characteristics of the included studies.

Study (Year)	Participants	Interventions	On/Off	Training Protocol	Follow-Up	Measured Outcomes and Tools	Main Findings
Brown et al. (2024) [27]	*n*: 14 IG 1: 7 IG 2: 7 Mean age: IG 1: 70 ± 7 IG 2: 69 ± 7 Sex (M%): IG 1: 57% IG 2: 42% H&Y: I–III Mean DD: IG 1: 8 ± 8 IG 2: 9 ± 7	IG 1: participants used a calibrated threshold for EMST IG 2: participants received therapeutic singing	On	IG 1 trained 5 days per week for 4 weeks They completed 5 sets × 5 reps IG 1 intensity was set at 75% MEP, and it was increased by a quarter each week IG 2 were given a homebased therapeutic singing protocol 5 days per week for 4 weeks IG 2 duration was 25 min	No	QoL: PDQ-39, PAS, GDS	QoL: No significant differences between groups
Antonsson et al. (2023) [28]	*n*: 19 (MS + PD) PD group: 9 IG: 5 CG: 4 Mean age (years (range)): 57.3 years Sex (M/F): 3/6 H&Y: I–III Mean DD: 5 years	IG: participants used a calibrated threshold for EMST CG: participants received sham treatment	NR	IG trained 5 days per week for 5 weeks They completed 25 reps and rest 15–30 s between breaths IG intensity was set at 75% MEP Homebased programme	No	Expiratory muscle strength: MEP Phonatory measures: MPT, DDK task, QASD	Expiratory muscle strength: Significant differences between baseline and post-EMST in IG. Phonatory measures: Significant differences between baseline and post-EMST for DDK. No significant differences between baseline and post-EMST for MPT and QASD
Troche et al. (2023) [29]	*n*: 58 IG 1: 30 IG 2: 28 Mean age (years (range)): IG 1: 70.5 IG 2: 69.1 Sex (M/F): IG 1: 21/13 IG 2: 22/9 H&Y: I–IV Mean DD: IG 1: 8 years IG 2: 7.6 years	IG 1: participants used a calibrated threshold for EMST IG 2: participants received a cough training approach called smTAP. They used a peak flow meter	On	All participants trained 5 days per week for 5 weeks They completed 5 sets × 5 reps IG: intensity was set at 75% MEP IG2: target set at 25% above baseline PEFR Homebased programme (1 supervised session once a week)	No	Expiratory muscle strength: MEP Cough volume: Voluntary CEV, Reflex CEV Peak flow: Voluntary PEFR, Reflex PEFR	Expiratory muscle strength: Significant differences between groups in favour of IG 1 Cough volume: Significant differences between groups in favour of IG2 Peak flow: Significant differences between groups in favour of IG 2
Mohammed et al. (2023) [30]	*n*: 18 IG 1: 9 IG 2: 9 Mean age: IG 1: 70.22 ± 6.18 IG 2: 69.67 ± 5.89 Sex (M/F): NR H&Y: I–III Mean DD: NR	IG 1: participants used an incentive spirometer IG 2: participants used a calibrated threshold for IMST	NR	All participants trained 6 days per week, 15 min twice a day, during 6 weeks IG 2 intensity was set at 0% MIP 5% increase every week	No	Inspiratory muscle strength: MIP Pulmonary fuction: FVC, FEV1 Peak flow: PEFR Exercise capacity: 6-MWT	Inspiratory muscle strength: Significant differences between groups in favour of IG 2 Pulmonary function: No significant differences between groups Peak flow: No significant differences between groups Exercise capacity: Significant differences between groups in favour of IG 2
Claus et al. (2021) [31]	*n*: 50 CG: 25 IG: 25 Mean age: CG: 67.1 ± 7.7 IG: 67.3 ± 9.5 Sex (M/F): CG: 19/5 IG: 18/5 H&Y: II–IV Mean DD: CG: 6.5 ± 7.7 IG: 6.6 ± 2.8	CG: participants received sham training IG: participants used a calibrated threshold for EMST	NR	All participants trained 5 days per week for 4 weeks They did 5 sets × 5 reps per day IG: EMST intensity was set at 75% MEP	3 months	Dysphagia symptoms: SDQ, FEES dysphagia score Cortical swallowing organisation: MEG Swallowing QoL: SWAL-QoL	Dysphagia symptoms: Significant differences between groups in favors to IG post-treatment as well as follow-up Cortical swallowing organisation: No significant differences between groups Swallowing QoL: No significant differences between groups.
Reyes et al. (2020) [32]	*n*: 31 CG: 10 IG 1: 10 IG 2: 11 Mean age: CG: 70.20 ± 6.69 IG 1: 70.45 ± 8.16 IG 2: 70.40 ± 6.81 Sex (M/F): CG: 4/6 IG 1: 6/5 IG 2: 7/3 H&Y: I–III Mean DD: NR	CG: participants used a threshold with fixed resistance (minimum pressure) IG 1: participants used a threshold with progressive resistance for EMST IG 2: participants used a threshold with progressive resistance for IMST	On	All participants trained 6 days per week for 8 weeks They did 5 sets × 5 reps per day Intensity from 50% to 75% MEP and MIP % adjusted every 2 weeks	No	Expiratory muscle strength: MEP Inspiratory muscle strength: MIP Phonatory measures: Mean SGP, MPT, Mean SPL Peak flow: Voluntary PCF	Expiratory muscle strength: Significant differences in effect size between IG1 and CG and also IG1 and IG2 Inspiratory muscle strength: Differences in effect size were moderate between IG2 and CG, small between IG2 and IG1 and also IG1 and CG Phonatory measures: Differences in effect size were trivial between IG2 and CG, small between IG1 and IG2 and moderate between IG1 and CG Peak flow: Differences in effect size were trivial between IG2 and CG, moderate between IG1 and IG2 and large between IG1 and CG
Reyes et al. (2018) [33]	*n*: 31 CG: 10 IG 1: 10 IG 2: 11 Mean age: CG: 70.20 ± 6.69 IG 1: 70.45 ± 8.16 IG 2: 70.40 ± 6.81 Sex (M/F): CG: 4/6 IG 1: 6/5 IG 2: 7/3 H&Y: I–III Mean DD: NR	CG: participants used a threshold with fixed resistance (minimum pressure) IG 1: threshold with progressive resistance for EMST IG 2: threshold with progressive resistance for IMST	On	All participants trained 6 days per week for 8 weeks They did 5 sets × 5 reps per day Intensity: from 50% to 75% MEP and MIP % adjusted every 2 weeks	No	Expiratory muscle strength: MEP Inspiratory muscle strength: MIP Pulmonary function: FVC, SVC Peak flow: Voluntary PCF, Reflex PCF	Expiratory muscle strength: Differences in effect size between IG2 and CG was moderate, large between IG1 and IG2 and between IG1. Inspiratory muscle strength: Differences in effect size between IG2 and CG was moderate, small between IG2 and IG1 and between IG1 and CG. Pulmonary function: Post-intervention effect size was very small in all groups. Peak flow: For Voluntary PCF, differences in effect size between IG2 and CG, moderate between IG1 and IG2, and large effect between IG1 and CG. Reflex peak cough flow had a trivial positive effect between IG2 and CG, a trivial negative effect between IG1 and IG2, and a moderate positive effect between IG1 and CG
Sapienza et al. (2011) [34]	*n*: 16 CG: 8 IG: 8 Mean age: CG: 68.50 ± 10.31 IG: 66.73 ± 8.90 Sex (M/F): CG: 22/8 IG: 25/5 H&Y: II–III Mean DD: NR	CG: participants received sham treatment IG: participants used a calibrated threshold for EMST	On	All participants trained 5 days per week for 4 weeks They did 5 sets × 5 reps IG intensity: NR Homebased programme	No	Expiratory muscle strength: MEP Pulmonary function: FVC, FEV1, FEV1/FVC Peak flow: PEFR	Expiratory muscle strength: Significant differences between groups in favour of IG Pulmonary function: No significant differences between groups Peak flow: No significant differences between groups
Troche et al. (2010) [35]	*n*: 60 CG: 30 IG: 30 Mean age: IG: 66.7 ± 8.9 CG: 68.5 ± 10.3 Sex (M/F): IG: 25/5 CG: 22/8 H&Y: II–IV Mean DD: NR	IG: participants used a calibrated threshold for EMST CG: participants received sham training	NR	All participants trained 5 days per week for 4 weeks They did 5 sets × 5 reps per day IG intensity was set at 75% MEP	No	Swallow safety: PA Score QoL: SWAL-QoL Duration of hyoid elevation: VFS Hyoid displacement: VFS	Sallow safety: Significant differences between groups in favour of IG Sallowing QoL: No significant differences between groups. Both groups improved. Duration of hyoid elevation: Significant impairment between baseline and post-sham in CG Hyoid displacement: Significant impairment between baseline and post-sham in CG
Inzelberg et al. (2005) [36]	*n*: 20 CG: 10 IG: 10 Mean age: CG: 65.2 ± 3.6 IG: 59.4 ± 2.4 Sex (M/F): IG: 9/1 CG: 9/1 H&Y: II–III Mean DD: IG: 8.58 ± 1.8 CG: 8.15 ± 2.0	IG: participants used a calibrated trheshold for IMST CG: participants received sham training	On	All participants trained 6 days per week for 12 weeks They did 30 min per day IG intensity was set from 15% to 60% MIP % weekly adjusted	No	Inspiratory muscle strength: MIP Pulmonary function: FVC, FEV1 Inspiratory muscle endurance: PmPeak POD: Borg Scale QoL: SF-36 questionnaire	Inspiratory muscle strength: Significant differences between groups in favour of IG Pulmonary function: No significant differences between groups Inspiratory muscle endurance: Significant differences between groups in favour of IG POD: Significant differences between groups in favour of IG QoL: No significant differences between groups

CG: control group, IG: intervention group, PD: Parkinson’s disease, MS: multiple sclerosis, M/F: male/female, H&Y: Hoehn and Yahr scale, DD: disease duration, NR: not reported, EMST: expiratory muscle strength training, IMST: inspiratory muscle strength training, MEP: maximum expiratory pressure, MIP: maximum inspiratory pressure, MPT: maximum phonation time, PDQ-39: Parkinson’s disease questionnaire-39, PAS: Parkinson’s Anxiety Scale, GDS: Geriatric Depression Scale, DDK: diadochokinetic rate, QASD: Questionnaire on Acquired Speech Disorders, smTAP: sensorimotor training for airway protection, PCF: peak cough flow, CEV: cough expiratory volume, 6-MWT: 6 min walking test, FVC: Force Vital Capacity, FEV1: forced expiratory volume in 1 s, PEFR: peak expiratory flow rate, SDQ: Swallowing Disturbance Questionnaire, FEES: fibreoptic endoscopic evaluation of swallowing score, MEG: magnetoencephalography studies, QoL: quality of life, SWAL-QOL: Swallowing Quality of Life Questionnaire, SPL: Sound Pressure Level, SGP: subglottic pressure, SVC: slow vital capacity, VFS: videofluoroscopic studies, PA Score: penetration–aspiration score, PmPeak: peak pressure, POD: perception of dyspnoea.

**Table 3:** Methodological quality of the included studies according to Downs and Black [28,29,30,31,32,33,34,35,36,37].

Author (Year)	Reporting	External Validity	Internal Validity (Bias)	Confounding and Selection Bias	Power	Total
Brown et al. (2024) [27]	8/11	1/3	6/7	3/6	0/1	18/28
Antonsson et al. (2023) [28]	10/11	3/3	6/7	6/6	0/1	25/28
Troche et al. (2023) [29]	9/11	2/3	7/7	5/6	1/1	24/28
Mohammed et al. (2023) [30]	8/11	1/3	5/7	3/6	1/1	18/28
Clauss et al. (2021) [31]	9/11	3/3	6/7	6/6	1/1	25/28
Reyes et al. (2020) [32]	9/11	3/3	6/7	4/6	1/1	23/28
Reyes et al. (2018) [33]	8/11	2/3	6/7	4/6	1/1	21/28
Sapienza et al. (2011) [34]	7/11	3/3	7/7	4/6	0/1	21/28
Troche et al. (2010) [35]	8/11	3/3	7/7	5/6	0/1	23/28
Inzelberg et al. (2005) [36]	8/11	0/3	7/7	3/6	0/1	18/28

## Data Availability

No new data were created or analysed in this study. Data sharing is not applicable to this article.

## Supplementary Materials

The following supporting information can be downloaded at https://www.mdpi.com/article/10.3390/healthcare13101214/s1, Supplementary File S1: PRISMA checklist.

## References

[1] Balestrino R., Schapira A. (2020). Parkinson disease. Eur. J. Neurol..

[2] World Health Organization WHO (2023). Parkinson Disease. https://www.who.int/news-room/fact-sheets/detail/parkinson-disease.

[3] Dorsey A., Constantinescu R., Thompson P., Biglan M., Holloway G., Kieburtz K., Marshall F.J., Ravina B.M., Schifitto G., Siderowf A. (2007). Projected number of people with Parkinson disease in the most populous nations, 2005 through 2030. Neurology.

[4] Politis M., Wu K., Molloy S., Bain P., Chaudhuri R., Piccini P. (2010). Parkinson’s disease symptoms: The patient’s perspective. Mov. Disord..

[5] Khoo K., Yarnall J., Duncan W., Coleman S., O’Brien T., Brooks J., Braker R.A., Burn D.J. (2013). The spectrum of nonmotor symptoms in early Parkinson disease. Neurology.

[6] De Souza S., Dionísio C., Almeida L. (2011). Multi-joint movements with reversal in Parkinson’s disease: Kinematics and electromyography. J. Electromyogr. Kinesiol..

[7] Sathyaprabha N., Kapavarapu K., Pal K., Thennarasu K., Raju R. (2005). Pulmonary functions in Parkinson’s disease. Indian J. Chest Dis. Allied Sci..

[8] Wang Y., Shao W.-B., Gao L., Lu J., Gu H., Sun L.-H., Tan Y., Zhang Y.-D. (2014). Abnormal pulmonary function and respiratory muscle strength findings in Chinese patients with Parkinson’s disease and multiple system atrophy–comparison with normal elderly. PLoS ONE.

[9] De Bruin F., de Bruin M., Lees A.J., Pride N.B. (1993). Effects of treatment on airway dynamics and respiratory muscle strength in Parkinson’s disease. Am. Rev. Respir. Dis..

[10] Polatli M., Akyol A., Çildaǧ O., Bayülkem K. (2001). Pulmonary function tests in Parkinson’s disease. Eur. J. Neurol..

[11] Ramig L.O., Fox C., Sapir S. (2008). Speech treatment for Parkinson’s disease. Expert. Rev. Neurother..

[12] Torsney K.M., Forsyth D. (2017). Respiratory dysfunction in Parkinson’s disease. J. R. Coll. Phys. Edinb..

[13] Baille G., Perez T., Devos D., Deken V., Defebvre L., Moreau C. (2018). Early occurrence of inspiratory muscle weakness in Parkinson’s disease. PLoS ONE.

[14] Haas B.M., Trew M., Castle P.C. (2004). Effects of respiratory muscle weakness on daily living function, quality of life, activity levels, and exercise capacity in mild to moderate Parkinson’s disease. Am. J. Phys. Med. Rehabil..

[15] Takizawa C., Gemmell E., Kenworthy J., Speyer R.A. (2016). Systematic review of the prevalence of oropharyngeal dysphagia in stroke, Parkinson’s disease, Alzheimer’s disease, head injury, and pneumonia. Dysphagia.

[16] Illi S.K., Held U., Frank I., Spengler C.M. (2012). Effect of respiratory muscle training on exercise performance in healthy individuals: A systematic review and meta-analysis. Sports Med..

[17] Aliverti A. (2020). Respiratory muscle training: Theory and practice. Breathe.

[18] Silva I.S., Pedrosa R., Azevedo I.G., Forbes A.M., Fregonezi G.A., Junior M.E.D., Lima S., Ferreira G. (2019). Respiratory muscle training in children and adults with neuromuscular disease. Cochrane Database Syst. Rev..

[19] Menezes K.K., Nascimento L.R., Ada L., Polese J.C., Avelino P.R., Teixeira-Salmela L.F. (2016). Respiratory muscle training increases respiratory muscle strength and reduces respiratory complications after stroke: A systematic review. J. Physiother..

[20] Zhuang J., Jia J. (2022). Effects of respiratory muscle strength training on respiratory-related impairments of Parkinson’s disease. Front. Aging Neurosci..

[21] Rodríguez M.Á., Crespo I., Del Valle M., Olmedillas H. (2020). Should respiratory muscle training be part of the treatment of Parkinson’s disease? A systematic review of randomized controlled trials. Clin. Rehabil..

[22] Page M.J., Moher D., Bossuyt P.M., Boutron I., Hoffmann T.C., Mulrow C.D., McKenzie J.E. (2021). PRISMA 2020 explanation and elaboration: Updated guidance and exemplars for reporting systematic reviews. BMJ.

[23] Centre for Reviews & Dissemination (2009). Systematic Reviews: CRD’s Guidance for Undertaking Systematic Reviews in Healthcare.

[24] Downs S.H., Black N. (1998). The feasibility of creating a checklist for the assessment of the methodological quality both of randomised and non-randomised studies of health care interventions. J. Epidemiol. Community Health..

[25] Higgins J.S.G. (2011). Cochrane Handbook for Systematic Reviews of Interventions Version 5.1.0.

[26] Higgins J., Thomas J., Chandler J., Cumpston M., Page M., Welch V. (2021). Cochrane Handbook for Systematic Reviews of Interventions Version 6.2.

[27] Brown J., Stegemöller E.L. (2024). Therapeutic singing and expiratory muscle strength training in Parkinson’s disease: A mixed methods comparison. Front. Rehabil. Sci..

[28] Antonsson M., Johansson K., Bonde-Dalemo A., Ivehorn-Axelsson C., Burge Å., Lesueur U., Hartelius L. (2023). Effect of expiratory muscle strength training on voice and speech: An exploratory study in persons with Parkinson’s disease or multiple sclerosis. Int. J. Speech-Lang. Pathol..

[29] Troche M.S., Curtis J.A., Sevitz J.S., Dakin A.E., Perry S.E., Borders J.C., Grande MPhil A.A., Mou Y., Vanegas-Arroyave N., Hegland K.W. (2023). Rehabilitating cough dysfunction in Parkinson’s disease: A randomized controlled trial. Mov. Disord..

[30] Mohammed-Yusuf S.F., Bhise A., Nuhmani S., Alghadir A.H., Khan M. (2023). Effects of an incentive spirometer versus a threshold inspiratory muscle trainer on lung functions in Parkinson’s disease patients: A randomized trial. Sci. Rep..

[31] Claus I., Muhle P., Czechowski J., Ahring S., Labeit B., Suntrup-Krueger S., Wiendl H., Dziewas R., Warnecke T. (2021). Expiratory muscle strength training for therapy of pharyngeal dysphagia in Parkinson’s disease. Mov. Disord..

[32] Reyes A., Castillo A., Castillo J., Cornejo I., Cruickshank T. (2020). The effects of respiratory muscle training on phonatory measures in individuals with Parkinson’s disease. J. Voice.

[33] Reyes A., Castillo A., Castillo J., Cornejo I. (2018). The effects of respiratory muscle training on peak cough flow in patients with Parkinson’s disease: A randomized controlled study. Clin. Rehabil..

[34] Sapienza C., Troche M., Pitts T., Davenport P. (2011). Respiratory strength training: Concept and intervention outcomes. Semin. Speech Lang..

[35] Troche M.S., Okun M.S., Rosenbek J.C., Musson N., Fernandez H.H., Rodriguez R., Sapienza C.M. (2010). Aspiration and swallowing in Parkinson disease and rehabilitation with EMST: A randomized trial. Neurololgy.

[36] Inzelberg R., Peleg N., Nisipeanu P., Magadle R., Carasso R.L., Weiner P. (2005). Inspiratory muscle training and the perception of dyspnea in Parkinson’s disease. Can. J. Neurol. Sci..

[37] Lauren H., Nathalie J., Alexandra F., Thomas S., Tamara P. (2016). The Incidence of Parkinson’s Disease: A Systematic Review and Meta-Analysis. Neuroepidemiology.

[38] Van de Wetering V., Kalf J.G., Van der Wees P.J., Bloem B.R., Nijkrake M.J. (2020). The effects of respiratory training in Parkinson’s disease: A systematic review. J. Park. Dis..

[39] Watson K., Egerton T., Sheers N., Retica S., McGaw R., Clohessy T., Webster P., Berlowitz D.J. (2022). Respiratory muscle training in neuromuscular disease: A systematic review and meta-analysis. Eur. Respir. Rev..

[40] Gauld L.M. (2009). Airway clearance in neuromuscular weakness. Dev. Med. Child. Neurol..

[41] Shapira N., Zabatino S.M., Ahmed S., Murphy D.M., Sullivan D., Lemole G.M. (1990). Determinants of pulmonary function in patients undergoing coronary bypass operations. Ann. Thorac. Surg..

[42] Romanini W., Muller A.P., Carvalho D., Olandoski M., Faria-Neto J.R., Mendes F.L. (2007). The effects of intermittent positive pressure and incentive spirometry in the postoperative of myocardial revascularization. Arq. Bras. Cardiol..

[43] Docu-Axelerad A., Stroe A.Z., Arghir O.C., Docu-Axelerad D., Gogu A.E. (2021). Respiratory dysfunctions in Parkinson’s disease patients. Brain Sci..

